# Re-look at socioeconomic inequalities in stroke prevalence among urban Chinese: is the inflexion approaching?

**DOI:** 10.1186/s12889-018-5279-y

**Published:** 2018-03-20

**Authors:** Shenghua Li, Fei Xu, Jing He, Zhiyong Wang, Lap Ah. Tse, Yaqing Xiong, Daowen Chen

**Affiliations:** 1 Department of Neurology, The Affiliated Jiangning Hospital of Nanjing Medical University, Nanjing, China; 2 Nanjing Municipal Center for Disease Control and Prevention, Nanjing, China; 3 Department of Epidemiology, School of Public Health, Nanjing Medical University, Nanjing, China; 4 School of Public Health and Primary Care, The Chinese University of Hong Kong, Hong Kong, China; 5 Jiangsu Province Geriatric Hospital, 30, Luojia Road, Nanjing, 210024 China; 6 Department of Neurology, The Affiliated Brain Hospital of Nanjing Medical University, 264, Guangzhou Road, Nanjing, 210029 China

**Keywords:** Stroke, Prevalence, Socioeconomic status, Family average income

## Abstract

**Background:**

The present association between socioeconomic status (SES) and stroke is positive in developing communities, but it is negative in developed countries where a positive SES-stroke relationship was recorded several decades ago. We hypothesized that the SES-stroke relationship in developing societies mirrors the trajectory of the Western countries at some stage of economic development. This study aimed to examine whether this inflexion is approaching in China.

**Methods:**

This study comprises of two cross-sectional surveys conducted in the same urban areas of Nanjing, China in 2000 (S2000) and 2011 (S2011) using the same selection criteria (i.e., aged≥35 years) and sampling approach. Physician-diagnosed stroke was the outcome event, while family average income (FAI) was the explanatory variable and tertiled in our anlaysis. Mixed-effects models were used to examine the FAI-stroke association.

**Results:**

Overall, 19,861 (response rate = 90.1%) and 7824 (response rate = 82.8%) participants participated in the S2000 and S2011, respectively. The prevalence of stroke increased by 2.5-folds (95%CI = 2.2, 2.9) from 2000 (2.1%, 95%CI = 1.9%, 2.3%) to 2011 (5.1%, 95%CI = 4.6%, 5.6%) (*p* < 0.01). Compared with the lower FAI category, the positive association between stroke prevalence and the higher FAI group decreased from 1.99 (95%CI = 1.55, 2.56) in 2000 to 1.49 (95%CI = 1.09, 2.03) in 2011 after control for potential confounders. A similar pattern was also observed for the middle FAI group (1.60, 95% CI = 1.23, 2.08 in 2000 vs. 1.37, 95%CI = 1.01, 1.88 in 2011).

**Conclusions:**

This study revealed that socioeconomic inequalities in stroke were diminishing in regional China during the recent 11-year period, although the SES-stroke association was still positive. Tailored intervention against stroke should currently target on SES-vulnerable people.

## Background

Inconsistent association between socioeconomic status (SES) and stroke prevalence has been reported worldwide, with various indicators of SEE in different studies [1–10]. Inflexional results were observed at different socioeconomic stages in Western countries, transiting from a positive association decades ago to a negative link in more recent years [1–10].

SES is a complex index and is commonly measured by residents’ income, educational attainment or occupation. Although different SES measures can provide different information on residents’ socioeconomic positions within their communities [11], family average income (FAI) has been shown to be a more sensitive indicator of SES as it can more realistically reflect material measures than educational attainment or occupation [12–14].

In China, it has been documented that the prevalence of stroke was positively associated with increasing levels of all SES indices, including family average income, educational attainment and occupation [6]. As China has experienced rapid economic development since the “Open Door” policy launched in 1978, the Gross Domestic Product (GDP) in 2015 is approximately 185.4 times high as that in 1978 based on the statistics of China currency [15]. We hypothesized that the relationship between SES and stroke in China mirrors the trajectory of that in the Western countries, with approximately 30 years lag as a result of economic growth and social development.

We adopted FAI as an indicator of SES in this study, aiming at examining whether there was an inflexion of the association between SES and stroke (i.e., transiting from a positive association to a negative one) by comparing representative data from surveys in 2000 and 2011 among Chinese adults in urban areas of China.

## Methods

### Participants and sampling

Two population-based cross-sectional surveys were conducted separately in 2000 and 2011 (hereafter, S2000 and S2011) in Nanjing, China. Nanjing is located in the east of China with a registered population of 5.6 million in 2000 and 6.4 million in 2011. Participants of these two surveys were randomly selected from the same urban areas of Nanjing. In China, the administrative system comprises of five strata: Central government, Provincial/Municipal government, District/County government, Street/Town Government and Administrative Village.

The same sampling approaches were adopted for the two surveys. First, targeted urban districts were randomly selected. Then, administrative streets were chosen from the selected districts. Next, administrative villages were selected from those chosen streets. Lastly, neighbourhoods were randomly selected from the selected administrative villages and all the eligible participants from those neighbourhoods were invited to take part in the survey. To be eligible, participants had to be local residents registered in the chosen neighbourhoods for at least 5 years and aged 35 years or above. Overall, these two surveys used the same inclusion criteria to recruit study participants.

Ethics approval was obtained separately for each survey from the Academic and Ethical Committee of Nanjing Municipal Center for Disease Control and Prevention. Informed consent was obtained from each participant prior to each survey.

### Sample size estimation

The prevalence of stroke used to estimate the sample size of our S2000 was 1.9% according to a population-based survey in urban areas of a neighbour city in 2000 [16]. Given this reference stroke prevalence of 1.9% in 2000 and assuming an absolute precision of 0.2%, the sample size of S2000 was estimated to be approximately 17,901. On the other hand, it was reported that the prevalence of stroke was 3.6% among a representative sample of local urban adults in 2007 [17]. Assuming an absolute precision of 0.4% and stroke prevalence of 3.6%, the required sample size for S2011 was calculated to be about 6987.

These statistics suggested that statistical power was adequate for achieving the research objectives of the current study. The actual number of participants for S2000 and S2011 were more than the required sample size which indicates that the statistical power for the two surveys was adequate for achieving the research objectives of our current study.

### Data collection and questionnaires

A standardized questionnaire was applied in the S2000 survey [18] to collect information on participants’ socio-demographic characteristics, stroke status, blood pressure status, diabetes status, number of family members, total monthly income of family, smoking status, alcohol drinking, and physical activity (PA), consumption of red-meat and healthcare payment. The same questionnaire was also used for the S2011 survey except for physical activity. In S2111, the validated Chinese version of International Physical Activity Questionnaire (IPAQ-CHIN) was used to collect information on PA [19]. In each survey, questionnaire was administered by well-trained interviewers.

### Specification of study variables

#### Outcome variable- stroke prevalence

Self-reported stroke was treated as outcome variable and categorized into: (1)“stroke” or (2)“non-stroke” in this study. Stroke was diagnosed based on the WHO MONICA criteria [20]. Each participant was invited to respond to the question “Have you ever been diagnosed as a stroke patient by a doctor or doctors at a hospital?” via face-to-face interview. If the answer was “Yes”, the participant/guardian was then asked to show the patient’s medical record to confirm his/her declaration. In China, each resident has a personal medical record with him−/her-self after he/she visits a doctor. Details about sub-types of stroke, date of diagnosis, evidence related to the diagnosis, hospital of diagnosis and the current medication status (if any) were extracted from the medical record. No medical record was traced if the participant answered “No” to the question about stroke. It was less likely that the participant forget/ignore experiencing a stroke event diagnosed by clinical doctors.

#### Explanatory variable-family average income

Family average income was used as the explanatory variable to indicate participant’s socio-economic status in this study. FAI was defined as the self-reported total monthly incomes of all family members divided by the total number of family members (including children and elderly), which was further tertiled as lower, middle and higher sub-group for analysis. FAI, as an individual level marker of SES, has been examined to be able to more sensitively and realistically reflect participants’ socioeconomic standing in the community than other indicators such as education or occupation [12–14], therefore FAI was chosen as the indicator of SES in this study. Family members referred to those who lived together and shared living-related expenditures. The total monthly income of all family members was the total monthly earnings of the whole family, including salaries, pensions and allowances, and money from selling goods and products.

#### Potential confounding factors

In our analysis, the available conventional stroke risk factors were treated as potential confounding factors, including age, gender, educational attainment, occupation, smoking, alcohol drinking, red-meat consumption, healthcare payment, diabetic status, blood pressure status, body weight status and leisure-time physical activity (LTPA). Socio-demographic covariates were categorized into: (1) younger (35–49 years), middle (50–64 years) or older (65+ years) for age; (2) men and women for gender; (3) 0–6 years, 7–12 years, or 13+ years based on years of schooling completed by participants for educational attainment; and (4) white collar and blue collar according to their workplaces (at office or not) for occupation.

Smoking status was classified as ‘smokers’ (those who smoked at least one cigarette per day continuously for at least one year, or smoked at least eighteen packs in total each year) and ‘non-smokers’ (those who had never smoked cigarettes, or smoked but did not meet the criteria of ‘smokers’), while alcohol drinking status was categorized into “drinkers” (those who drank alcohol two or more times per week on average, for at least 1 year) and “non- drinkers” (those who never drank or drank alcohol but did not meet the criterion of regular drinkers).

Red-meat consumption was tertiled as lower, middle and upper group based on the distribution of weekly frequency of consumption [21]. The information on weekly consumption of red meat was gathered using a validated food frequency questionnaire [22]. Healthcare payment was categorized as “Insured” (by employers) and “Non-insured” (healthcare paid by selves). Healthcare payment referred to how the healthcare fees were paid. Diabetic status was grouped as “Yes” or “No” according to the diagnostic information from the personal medical record. The status of blood pressure was categorized as “Hypertension” and “Non-hypertension”. Hypertension was diagnosed for a participant by registered physicians if either the systolic blood pressure exceeded 140 mmHg or the diastolic blood pressure exceeded 90 mmHg, or both, with consideration of clinical symptoms. Moreover, those participants who have prescribed to take anti-hypertension medications were also defined as hypertensive patients even if their blood pressure was normal when measured.

Body weight status was classified as “Normal weight” and “Excess weight” based on the recommended cutoffs of body mass index (BMI) [weight (kg)/height (m^2^)] for Chinese adults. Excess weight was defined as BMI equal to or greater than 24, while non-excess weight as BMI less than 24. [23] Height and weight were objectively measured twice at each visit and the mean of the two readings was used in the analysis.

Leisure-time physical activity was classified as “Sufficient (the sum of moderate-intensity and doubled vigorous-intensity PA time ≥ 150 minutes/week)” and “Insufficient (those not meet the above mentioned criteria of “Sufficient” PA)” according to existing recommendations [24]. The information on LTPA (type of intensity, weekly frequency and duration of each PA session) in the past week was collected from each participant, although different instruments were used in the two surveys. In S2000, LTPA was assessed with a widely-accepted questionnaire in which two likert items were used. The first item was “In last week, what type of LTPA you engaged in (No LTPA, Light, Moderate or Vigorous intensity)?”. The answer was “No" if the participant did not engage in LTPA in last week. If the answer was either light, moderate or vigorous intensity, the next question was "How many times you engaged in such LTPA separately (at least 15 min for each session) in last week (once, 2, 3, or 5 times per week)?”. For the S2011, LTPA was measured using the validated Chinese short-version of the International Physical Activity Questionnaire (IPAQ-CHN) [19].

### Data analysis

Chi-square test was calculated to compare the categorical socio-demographic characteristics of participants. Mixed-effects models were performed separately to compute the odds ratios (ORs) and 95% confidence intervals (95%CI) for the relationship between FAI and stroke based on two models. Model 1 was a univariate analysis with only FAI as the single predictor; Model 2 was a multivariate analysis with adjustment for conventional risk factors of stroke, including age, gender, educational attainment, occupation, smoking, alcohol drinking, red-meat consumption frequency, healthcare insurance, diabetes, status of blood pressure, body weight status, leisure-time physical activity and survey area (potential clustering by administrative unit). *p* < 0.05 was used to assess the two-side statistical significance. Data analyses were conducted using SPSS 21.0 (IBM Corp, Armonk, NY, USA).

## Results

### Socio-demographic characteristics

There were 19,861 (response rate = 90.1%) and 7824 (response rate = 82.8%) adults completing the S2000 and S2011 survey, respectively. Table 1 displays the selected socio-demographic characteristics in both surveys. Compared with the S2000, participants in the S2011 were significantly elder and less educated, but more white-collar workers and insured healthcare (71.9% in 2000 vs. 75.3% in 2011). Mean value of FAI was significantly increased by 2.9 times from 2000 to 2011 among our survey participants (*p* < 0.001). There was no significant difference in socio-demographic characteristics between respondents and non-respondents in each survey.

**Table 1:** Selected socio-demographic characteristics of participants in survey 2000 and 2011 in Nanjing, China

Variables	Survey 2000 (*N* = 19,861)	Survey 2011 (*N* = 7824)	*p* value^b^
	% (n)	% (n)	
Age (yrs)			
35–49	44.3 (8793)	40.3 (3154)	< 0.01
50–64	34.5 (6849)	34.0 (2662)	
65+	21.2 (4219)	25.7 (2008)	
Gender			
Female	50.3 (9990)	49.4 (3867)	0.68
Male	49.7 (9871)	50.6 (3957)	
Educational attainment (yrs)			
0–9	54.6 (10849)	59.8 (4681)	< 0.01
10–12	25.2 (4996)	24.6 (1928)	
13+	20.2 (4016)	15.5 (1215)	
Occupation^a^			
Blue collar	51.2 (10178)	49.7 (3889)	0.01
White collar	48.8 (9683)	50.3 (3935)	
Healthcare insurance			
Insured	71.9 (14282)	75.3 (5894)	< 0.01
Non-insured	28.1 (5579)	24.7 (1930)	
FAI (¥/m)	551.3 ± 426.7	1575.6 ± 1243.2	< 0.001

^a^Blue collar = farmer, factory worker, forestry worker, fisher and military person, salespeople, house worker and vehicle driver; White collar = office worker, teacher, doctor and academic researcher, government officer

^b^Chi-square test was calculated to compare the categorical socio-demographic characteristics of participants

There were 44.3% vs. 40.3%, 34.5% vs. 34.0% and 21.2% vs. 25.7% of participants within each age-group (35–49, 50–64 and 65+) for S2000 vs. S2011 (*p* < 0.05). This was in line with the local census data that the proportion of residents aged 65+ years old within general population significantly increased from16.5% in 2000 to 17.7% in 2011 (*p* < 0.05). The male to female ratio in S2000 (1:1.01) and S2011 (1:1.02) was comparable to that of the census data for the corresponding year (*p* > 0.05).

### Prevalence of self-reported stroke

Table 2 shows the prevalence of self-reported stroke among participants in S2000 and S2011, respectively. The prevalence of self-reported stroke was 2.1% (95%CI = 1.9%, 2.3%) in 2000, while it increased to 5.1% (95%CI = 4.6%, 5.6%) in 2011 with an increasing rate of 2.5-folds (95%CI = 2.2, 2.9) (*p* < 0.01). Although the prevalence of stroke was the smallest amongst the five major defined chronic diseases (0.1% in 2000 and 0.7% in 2011), the speed of increase was the fastest among younger age-group (4.5 times [95%CI = 2.3, 9.0] increase) compared to the middle-aged (2.6 times[95%CI = 2.0,3.4]) or older (2.1 times [95%CI = 1.7, 2.5]) group of participants (*p* = 0.04). From 2000 to 2011, there were 2.4 [95%CI = 2.0, 2.9] and 2.6 [95%CI = 2.1, 3.2] times increase in stroke prevalence among men and women (*p* = 0.66), and it increased 2.5[95%CI = 2.2, 2.9] and 2.2[95%CI = 1.6, 3.2] times among those with and without insured healthcare, respectively, (*p* = 0.51).

**Table 2:** Prevalence of self-reported stroke by age and gender among urban residents in 2000 and 2011 in Nanjing, China

Variables	Prevalence of self-reported stroke (%, n)	
	Survey 2000 (*N* = 19,861)	Survey 2011 (*N* = 7824)
Overall		
	2.1 (416)	5.1 (400)
Age (yrs)		
35–49	0.1 (13)	0.7 (21)
50–64	1.8 (125)	4.7 (124)
65+	6.6 (278)	12.7 (255)
Gender		
Women	1.7 (171)	4.3 (168)
Men	2.5 (245)	5.9 (232)
Education (yrs)		
0–9	2.1 (232)	5.7 (267)
10–12	1.4 (70)	4.2 (81)
13+	2.8 (114)	4.3 (52)
Occupation†		
Blue collar	0.9 (91)	2.3 (88)
White collar	3.4 (325)	7.9 (312)
Healthcare payment		
Insured	2.4 (342)	5.8 (344)
Non-insured	1.3 (74)	2.9 (56)

†Blue collar = farmer, factory worker, forestry worker, fisher and military person, salespeople, house worker and vehicle driver; White collar = office worker, teacher, doctor and academic researcher, government officer

### Relationship between FAI and stroke

Table 3 presents the relationship between FAI and stroke in S2000 and S2011, separately. Compared with the lower FAI category, the positive association of stroke prevalence with the high and middle FAI group decreased from 1.99 (95%CI = 1.55, 2.56) and 1.60 (95% CI = 1.23, 2.08) in S2000 to 1.49 (95%CI = 1.09, 2.03) and 1.37 (95%CI = 1.01, 1.88) in 2011, after control for potential confounders. Interestingly, with consideration of potential confounders, the size of the disparities of stroke prevalence within FAI categories attenuated significantly among overall participants (*p* = 0.03) over the 11 years. Furthermore, after stratified analysis, such significant attenuations were observed in women (*p* = 0.01) and among those middle-aged (*p* = 0.04) and elders aged 65+ years old (p = 0.01).

**Table 3:** The relationship between family average income and self-reported stroke prevalence in 2000 and 2011 among urban residents in Nanjing, China

		The S2000 study			The S2011 study			P value for comparison of ORs between S2000 and S2011^b^	P value for comparison of Adjusted ORs between S2000 and S2011^b^
Variables	FAI Category	Stroke (% and n)	Unadjusted Odds ratio (95%CI)	Adjusted Odds ratio^a^ (95%CI)	Stroke (% and n)	Unadjusted Odds ratio (95%CI)	Adjusted Odds ratio^a^ (95%CI)		
Overall									
	Lower	1.1 (79)	1	1	3.5 (103)		1	0.022	0.031
	Middle	2.3 (144)	2.06 (1.57,2.72)	1.46 (1.07,1.97)	5.5 (130)	1.60 (1.23,2.08)	1.37 (1.01,1.88)		
	Higher	2.9 (193)	2.65 (2.04,3.45)	1.66 (1.19,2.32)	6.7 (167)	1.99 (1.55,2.56)	1.49 (1.09,2.03)		
Age (yrs)									
35–49									
	Lower	0.3 (10)	1	1	0.6 (8)		1	0.580	0.448
	Middle	0.1 (3)	0.41 (0.11,1.50)	0.34 (0.09,1.36)	0.6 (5)	1.02 (0.33,3.14)	1.33 (0.41,4.34)		
	Higher	0.0 (0)	0	0	0.9 (8)	1.59 (0.60,4.25)	2.17 (0.69,6.81)		
50–64									
	Lower	1.2 (23)	1	1	3.7 (31)		1	0.041	0.043
	Middle	2.2 (50)	1.87 (1.14,3.07)	1.51 (0.89,2.56)	5.0 (48)	1.37 (0.87,2.18)	1.18 (0.69,2.01)		
	Higher	2.0 (52)	1.69 (1.03,2.78)	1.41 (0.79,2.52)	5.3 (45)	1.46 (0.91,2.33)	1.16 (0.66,2.03)		
65+									
	Lower	3.3 (46)	1	1	9.1 (64)		1	0.004	0.013
	Middle	6.9 (91)	2.19 (1.52,3.15)	1.69 (1.13,2.52)	13.8 (77)	1.60 (1.13,2.28)	1.50 (0.99, 2.29)		
	Higher	9.4 (141)	3.05 (2.17,4.28)	2.21 (1.43,3.42)	15.2 (114)	1.80 (1.30,2.49)	1.58 (1.06,2.35)		
Gender									
Women									
	Lower	0.9 (32)	1	1	3.1 (45)		1	0.014	0.011
	Middle	1.9 (58)	2.15 (1.39,3.32)	1.88 (1.17,3.03)	4.1 (52)	1.33 (0.89,2.00)	1.39 (0.85,2.26)		
	Higher	2.5 (81)	2.88 (1.91,4.35)	2.21 (1.30,3.74)	6.1 (71)	2.02 (1.38,2.96)	1.74 (1.06,2.84)		
Men									
	Lower	1.4 (47)	1	1	3.8 (58)		1	0.032	0.052
	Middle	2.7 (86)	1.97 (1.38,2.82)	1.21 (0.82,1.81)	7.0 (78)	1.88 (1.33,2.67)	1.33 (0.88,2.00)		
	Higher	3.4 (112)	2.46 (1.74,3.47)	1.36 (0.88,2.11)	7.2 (96)	1.95 (1.40,2.73)	1.23 (0.82,1.85)		

^a^Mixed-effects logistic regression models were used to calculate odds ratios with adjustment for age, gender, education, occupation, healthcare payment, BMI, smoking, alcohol drinking, red-meat consumption, diabetes, high blood pressure, leisure-time physical activity and survey area (potential clustering effects at survey area level)

^b^Logistic regression models were used to make comparison of ORs between the two studies, with stroke as the outcome variable, FAI, survey (S2000/S2011) and the FAI-by-survey interaction term as the explanatory variables. For the comparison of Adjusted ORs between tow studies, all related potential confounders were controlled in analysis

## Discussion

This study demonstrated a 2.4 times increase in the prevalence of self-reported stroke among urban Chinese adults aged 35 years old or above from 2000 to 2011. Inequalities in SES and stroke prevalence among urban Chinese had attenuated significantly over the 11-year period, despite the association between FAI (a more sensitive indicator of SES) and stroke was still positive. These findings suggested that the SES-stroke patterns in China nowadays were still similar to those in the Western countries approximately 30 years ago (positive association) [1–10] but the strength of such a positive association has been significantly weakening, which might imply that an inflexion of direction of SES-stroke relationship may be approaching in urban Chinese as a consequence of continuing economic growth and social development.

SES can not only simply predict participants’ social standing but also exert an important influence on experience of stroke. Currently, the patterns of relationship between SES and stroke were different in societies with different economic and social development stages - being negative in developed societies but positive in developing communities [25]. Given that the economic and social development goes on in China and the direction of association between SES and stroke has been changed from being positive at earlier development stage several decades ago to being negative at the present developed stage in developed countries, it is expected that such a pattern in China may gradually transit from being positive to negative in th future.

Regardless of the direction of its association with stroke, SES is a valuable determinant of stroke at population level worldwide. It is possible to optimize the performance-investment ratio for stroke prevention programs only if those people with vulnerable SES are identified and can receive more precise prevention approaches as early as possible. Therefore, it is of realistic meaningfulness for population-based stroke precise prevention to continuously examine the relationship between SES and stroke at different economic development stages for different communities. This is of particular importance for developing countries, as their economy is less developed and the subsequent transitions may involve in changing in both socioeconomic and lifestyle related factors.

Although it is not well understood how the specific SES can influence stroke, there are several potential mechanisms attributable to inequalities in socioeconomic determinants to health. These can be explained by people with different SES may have different lifestyles, behavioural patterns, psychosocial stress, access to healthcare and material circumstances [1, 5, 26, 27]. Among those potential determinants, psychosocial factors and healthcare access could explain only a very small portion of SES difference in stroke prevalence, while conventional lifestyle and behavioural risk factors were the main driven power for the disparities of stroke prevalence in people with different SES [7, 20]. Dietary and physical activity patterns and anthropometric scenarios vary among people with the same SES in developed and developing communities. It is well known that SES was negatively associated with the consumption of high-dense energy food, insufficient PA and being obese in developed countries [28, 29], while the scenarios were completely inverse in developing communities [30, 31]. This means that residents within lower SES stratum were less likely to consume high-dense energy foods and to be obese, but more likely to engage in sufficient PA than those with higher SES in developing societies, as is the case in the current context of China. Such disparities in the distribution of conventional risk factors in developed and developing communities might, at least partially, act as intermediates for the influence of SES on the development of stroke.

China has been experiencing a rapid economic growth over the past decades [15] and certainly has been seeing a continuous change in lifestyle and behavior patterns. Chinese people tended to consuming more high-dense energy foods and doing less physical activity than those were done decades ago [32, 33], which suggested that lifestyle and behavioral patterns have been Westernizing in China. Therefore, it is foreseeable that the direction of association between SES and stroke in China is prone to being similar to that in the Western countries. It is of interest to know at what stage of economic development that the direction of FAI-stroke association will change in China and when the critical time of the diminishing disparity between FAI and stroke comes before the presence of a negative relationship. This is of particular public health importance for developing population-level stroke prevention programs in China, as the SES-related vulnerable sub-population for stroke will be different at different economic development stage in a society.

There are several strengths of this study. First, representative participants were randomly selected for each survey from the same urban areas of Nanjing. Second, sufficient cases of outcome event were guaranteed by large sample size of each survey. Third, the two surveys were led by the same principle investigator, which implied that the study qualities would be under control for each survey. Fourth, the same instrument was used in the two surveys. Fifth, FAI, a sensitive SES indicator, was used to represent SES. Finally, conventional confounders were sufficiently considered in our analysis.

This study also has several limitations. (1) The outcome event was the diagnosed stroke, which may cause stroke prevalence under-estimated, although strong agreement has been documented between self-reported stroke cases and those were confirmed by medical record review or CT scan [34, 35]. (2) Family incomes were also self-reported. This may result in potentially under-estimating incomes, as Chinese people tend to under-report their earnings [36]; however, such misclassification on FAI is likely non-differential, which would lead the effect size to be under-estimated. (3) The cross-sectional design of these two surveys did not allow us inferring causality for the SES and stroke in this study. However, this study also disclosed relations between socioeconomic determinants and stroke prevalence which provides important message for policy prevention and medical resources’ allocation for the existing cases. (4) We measured the SES-stroke association at individual level without including a macro-level of social determinants such as policy; however, policy implementation within a same urban area was similar and thus it was not a major concern. (5) The influence of inflation on FAI was not considered (Consumer Price Index in 2011 was 1.28 times that in 2000 in Nanjing) [37], which might affect the comparability of FAI overtime, but this was not be a major concern as we only compared the contemporized pattern of FAI with stroke.

## Conclusions

Socioeconomic inequalities in stroke prevalence among urban Chinese were diminishing, despite the association between SES and stroke was still positive. This inflexion tendency was prominent in women and those people aged 50+ years old. Presently, policy and individual intervention tackling socioeconomic inequalities should target on the low income families who have increased stroke prevalence. Further reduction of socioeconomic inequalities in stroke prevalence among all Chinese is a national goal for the improvement of public health.

## Abbreviations

BMI: Body mass index
CI: Confidence intervals
FAI: Family average income
GDP: Gross Domestic Product
IPAQ: International Physical Activity Questionnaire
IPAQ-CHN: Chinese version of the International Physical Activity Questionnaire
LTPA: Leisure-time physical activity
ORs: Odds ratios
PA: Physical activity
SES: Socioeconomic status
WHO: World Health organization
